# Vector-Borne Zoonotic Lymphadenitis—The Causative Agents, Epidemiology, Diagnostic Approach, and Therapeutic Possibilities—An Overview

**DOI:** 10.3390/life14091183

**Published:** 2024-09-19

**Authors:** Martina Oršolić, Nikolina Sarač, Mirjana Balen Topić

**Affiliations:** 1 University Hospital for Infectious Diseases “Dr. Fran Mihaljević”, Mirogojska 8, 10 000 Zagreb, Croatia; mkomsic@bfm.hr (M.O.); nbogdanic@bfm.hr (N.S.); 2 School of Medicine, University of Zagreb, Šalata 3, 10 000 Zagreb, Croatia

**Keywords:** vector-borne, zoonosis, lymphadenitis, ticks, arthropods, diagnosis, therapy

## Abstract

In addition to common skin pathogens, acute focal lymphadenitis in humans can, in rare cases, be caused by a zoonotic pathogen. Furthermore, it can develop in the absence of any direct or indirect contact with infected animals, in cases when the microorganism is transmitted by a vector. These clinical entities are rare, and therefore often not easily recognized, yet many zoonotic illnesses are currently considered emerging or re-emerging in many regions. Focal zoonotic vector-borne lymphadenitis and its numerous causative agents, with their variegated clinical manifestations, have been described in some case reports and small case series. Therefore, we summarized those data in this narrative overview, with the aim of raising clinical awareness, which could improve clinical outcomes. This overview briefly covers reported pathogens, their vectors and geographic distribution, and their main clinical manifestations, diagnostic possibilities, and recommended therapy. Vector-borne tularemia, plague, bartonellosis, rickettsioses, borreliosis, and Malayan filariasis are mentioned. According to the existing data, when acute focal bacterial vector-borne zoonotic lymphadenitis is suspected, in severe or complicated cases it seems prudent to apply combined aminoglycoside (or quinolone) plus doxycycline as an empirical therapy, pending definite diagnostic results. In this field, the “one health approach” and further epidemiological and clinical studies are needed.

## 1. Introduction

Acute focal lymphadenitis is usually an infectious inflammation of one or more lymph nodes in one anatomic region. When lymph nodes are palpable beneath the skin it can manifest as a combination of symptoms including enlargement, tenderness and pain of lymph nodes, redness of the overlying skin, and, in most cases, fever. With the very rare exception of direct inoculation of microorganisms into the lymph node by trauma, diagnostic puncture, biopsy, or surgical procedures, the majority of lymphadenitis reflects infection of the tissues drained by regional lymphatic vessels. This clinical entity can be caused by bacteria, viruses, spirochetes, fungi, or parasites [1]. In the past, *Staphylococcus* spp., *Streptococcus* spp., and *Mycobacterium* spp. were the primary infectious agents commonly found in lymph nodes. However, with the use of new diagnostic tools, new emerging microorganisms responsible for lymphadenitis have been recognized, including zoonotic pathogens [2]. Microorganisms causing focal lymphadenitis usually enter through mucous membranes or damaged skin directly, originating from respiratory tract aerosol or droplets of infected persons or animals (i.e., streptococcal infections, tuberculous and nontuberculous mycobacterial infections, cutaneous form of *Corynebacterium diphtheriae* infection), originating from a contaminated environment (i.e., staphylococcal infection, cutaneous anthrax), or originating from urogenital excretions (genital HHV infection, syphilis, lymphogranuloma venereum, chancroid, HIV). Focal palpable lymphadenitis in humans can also develop after consuming contaminated food (tularemia or brucellosis) or may be caused by actinomycetes. Actinomycetes are normally considered noninvasive commensal members of the microbiota, but can also cause local infection after a disruption of mucous membrane continuity. However, focal lymphadenitis can have a zoonotic origin and can develop after animal scratches, bites, or after direct or indirect contact with animals (i.e., bartonellosis (cat-scratch disease) [3], brucellosis [4,5], *Capnocytophaga canimorsus* [6,7], *Pasteurella multocida* [8], and *Corynebacterium pseudotuberculosis* infection) [9]. Furthermore, the microorganisms causing focal lymphadenitis could be introduced to the human body by arthropod vectors (i.e., *Yersinia pestis*, *Francisella tularensis*, *Bartonellae*, *filariae*, *Borrelia* spp., *Rickettsiae*).

Differential diagnosis of multifocal/generalized lymphadenitis is even broader. The most common infectious causes are Epstein–Barr virus (EBV, currently HHV-4), Cytomegalovirus (CMV, currently HHV-5) and HIV infection, toxoplasmosis, brucellosis, secondary syphilis, disseminated histoplasmosis, mycobacterial infection, and cryptococcosis [10,11].

In rare cases of focal lymphadenitis, the patient claims a local identified or unidentified arthropod bite. Also, in some cases, there is a small bite wound or eschar found in a local lymphatic drainage region, suggesting an arthropod vector bite, in a patient who cannot recall an arthropod bite. Since the local skin mark can be nonspecific, it could be easily substituted for a nonspecific wound, and empirical antibiotic therapy can easily be mistakenly pointed towards common bacterial skin pathogens.

In general, it has been assumed that 60% of all human infectious diseases originated from animals, and even in circumstances of the COVID-19 pandemic, according to the European Centre for Disease Prevention and Control (ECDC) data, an increased number of reported cases of zoonotic illnesses was observed in 2021 compared to the year before [12,13].

In the case of zoonotic infection transmitted by arthropod vector, the spatial (geographical) and temporal relationship between the affected human and infected animal reservoir is blurred, which makes establishing the diagnosis more difficult. The presumably low overall incidence of vector-borne zoonotic lymphadenitis, or the introduction of the disease by travelers, migrants, migrating infected animals, or vectors from endemic to low- or zero-incidence regions, may cause a delay in diagnosis and could have a negative impact on the disease outcome. Especially in cases of highly lethal and highly infectious causative agents (i.e., *Yersinia pestis*, *Francisella tularensis*), the elevated level of clinical suspicion is crucial. To increase the awareness, in this review we attempt to explore the data on possible vector-borne zoonotic focal lymphadenitis pathogens, describe the epidemiology and the spectrum of possible clinical manifestations, summarize diagnostic approaches according to the pathogen, and suggest therapeutic options.

## 2. Tularemia

Tularemia is a serious, potentially life-threatening zoonotic disease also known as Francis disease, Pahvant Valley plague, deer-fly fever, rabbit fever, market men disease, water-rat trappers’ disease, wild hare disease (yato-byo), and Ohara disease [14].

### 2.1. Microbiology and Epidemiology

The causative agent of the disease is *Francisella tularensis*, a small, aerobic, catalase-positive, pleomorphic, Gram-negative coccobacillus, nonmotile, non-spore-forming, facultative intracellular pathogen that may be easily disseminated with a lethal dose of less than 10 organisms [14]. The genus *Francisella* includes four species that are potential human pathogens: *F. tularensis*, *F. philomiragia*, *F. hispaniensis*, and *F. opportunistica*. *F tularensis* is divided into four subspecies: *F. tularensis* subsp. *tularensis* (*F. tularensis* type A), *F. tularensis* subsp. *holarctica* (*F. tularensis* type B), *F. tularensis* subsp. *mediasciatica*, and *F. tularensis* subsp. *novicida*. *F. tularensis* subsp. *tularensis* (type A strains) and *F. tularensis* subsp. *holarctica* (type B strains) are responsible for most human cases (Table 1). Human disease is rarely associated with the other subspecies [15,16]. Tularemia occurs only in the Northern hemisphere. Type A strains are found in North America and rarely in Europe. Type B strains are found predominantly in Asia, Australia, and Europe but also in North America and are less virulent in humans [14,17]. According to the ECDC, there were 620 confirmed cases of human tularemia in 2022, and this was a decrease of 29.5% compared with the number of confirmed cases in 2021 [18]. More than 70.0% of cases were reported by Finland, France, Germany, and Sweden. A total of 475 tularemia cases (76.6%) were reported to have been acquired in the EU. Austria, France, Germany, Poland, and Sweden reported travel-associated cases. Only one case (0.2%) was imported from outside the EU (from Egypt), whereas for 144 cases (23.2%), there were no data on travel or the country of infection. Tularemia cases mainly occurred from July to November, but cases were observed all throughout the year. In 2022, infections peaked in the end-of-summer and early autumn months [18]. The organism infects a wide variety of wild and domestic vertebrates and invertebrates. In Europe, important mammals associated with *F. tularensis* infection include hares, hamsters, mice, rats, beavers, lemmings, and voles (Table 1). Transmission of *F. tularensis* to humans occurs most often by direct animal contact, indirect contact with contaminated animal products, or through the bite of an insect (ticks, mosquitoes, biting flies, horse flies, fleas, and lice). In the United States, the most commonly reported tularemia tick vectors include *Amblyomma americanum*, *Dermacentor andersoni*, *Dermacentor occidentalis*, and *Dermacentor variabilis*. In Europe, *Dermacentor reticulatus*, *Haemaphysalis concinna*, and *Ixodes ricinus* are most frequently associated [14]. A recent review, which considered the vector role of mosquitoes for *F. tularensis*, found that there is no conclusive evidence that mosquitoes can transmit *F. tularensis* during blood feeding. Based on that, it is most likely that mosquitoes are mechanical vectors, although further studies are needed [19]. Other routes of transmission include aerosol droplets, contact with contaminated water or mud, and animal bites [14]. Also, inoculation from freshwater fishhook injury has been described as like transmissions during an autopsy and from solid organ transplantation [20,21,22,23]. *F. tularensis* is highly contagious for humans and because of the potential danger to laboratory workers they should be warned when tularemia is suspected so they can manipulate specimens using Biosafety Level 3 practices, especially during procedures that might produce aerosols or droplets [24,25]. Human-to-human spread does not occur [26].

### 2.2. Pathogenesis

Infection occurs when *F. tularensis* penetrates through skin and mucosa disruption sites that may be inapparent, most often the conjunctival sac or oropharyngeal mucosa. The organism can also penetrate intact skin directly or be introduced through the skin by an arthropod vector bite. The infectious dose in humans depends on the site of inoculation. When inoculation occurs through the skin, mucous membranes, or when inhaled, the infectious dose is 10 to 50 organisms. When ingested, 10^6^ to 10^8^ organisms are required. After cutaneous inoculation, in the first 3–5 days *F. tularensis* multiplies at the site of inoculation and produces a papule. Ulceration may begin 2 to 4 days later. After multiplying at the site of inoculation it spreads to the regional lymph nodes and then may spread systemically by a lymphohematogenous route and involve multiple organs [14,27,28]. Infection with *F. tularensis* is characterized by an acute inflammatory response at sites of inoculation and involves fibrin, neutrophils, macrophages, and T lymphocytes that result in tissue necrosis. As the area of necrosis expands, thrombosis of adjacent arteries and veins may occur. Characteristics of tularemia are usually small, sometimes confluent granulomatous foci, which may caseate and be mistaken for tuberculosis. In some cases, necrotic foci may coalesce to form abscesses [29].

### 2.3. Clinical Manifestation

Clinical manifestation of *F. tularensis* infection can range from asymptomatic or inconsequential illness to acute sepsis and rapid death. This depends on the virulence of the particular organism, the portal of entry, the extent of systemic involvement, and the immune status of the host. Approximately 3 to 5 days (range 1 to 21 days) following exposure, nonspecific systemic symptoms of tularemia start abruptly and include fever, chills, anorexia, malaise, headache, fatigue, cough, myalgias, chest discomfort, vomiting, sore throat, abdominal pain, and diarrhea. Fever classically lasts for several days, but without treatment fever lasts for an average of 32 days, while chronic fatigue, weight loss, and lymphadenopathy may persist for many months longer. Less virulent strains cause a milder, self-limited illness that may resolve without therapy [14,30]. Depending on the portal of entry there are six major clinical forms of tularemia: ulceroglandular, glandular, oculoglandular, pharyngeal (oropharyngeal), pneumonic, and typhoidal (Table 2) [31].

**Figure 1:**
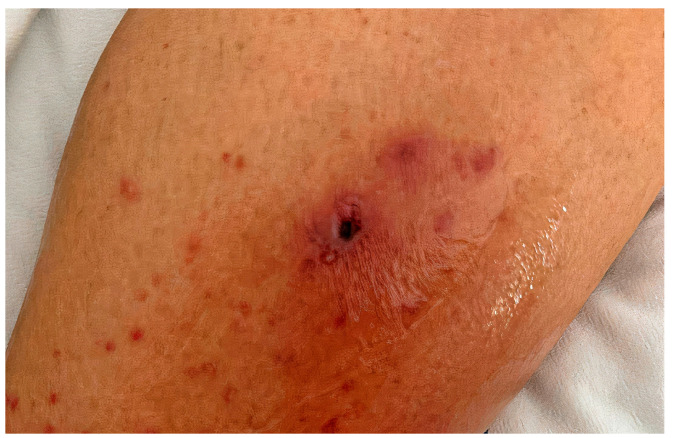
Local eschar on a lower leg skin in a patient with tularemia diagnosed by serology and molecular methods, who developed subsequently extensive purulent inguinal lymphadenitis.

Ulceroglandular disease is the most common clinical form of tularemia. Patients usually report a recent tick bite or animal contact. Patients with typical clinical presentation have fever and a single erythematous papulo-ulcerative lesion with a central eschar at the site of inoculation (Figure 1).

They also have enlarged and tender regional lymphadenopathy, which can occur before, at the same time, or shortly after the appearance of the skin lesion. Cervical and occipital lymphadenopahty are most common in children, while inguinal lymphadenopathy is the most common among adults. Skin changes over the involved nodes suggests underlying suppuration. Suppuration of affected lymph nodes is a relatively common complication and may occur despite antibiotic therapy in either ulceroglandular or glandular tularemia. Glandular tularemia occurs when patients present with tender regional lymphadenopathy but in the absence of a visible cutaneous lesion [14,30,31,32]. Oculoglandular tularemia occurs in a minority of cases due to the entrance of *F. tularensis* through the conjunctiva. Eye symptoms include pain, photophobia, and increased lacrimation. Symptoms are usually unilateral but may be bilateral, which is uncommon. Examination may show conjunctival erythema with edema, conjunctival purulence, small conjunctival ulcers or nodules, and periorbital erythema and/or edema. Tender regional lymphadenopathy may occur. Complications include corneal ulceration, dacryocystitis, nodal suppuration, and, rarely, vision loss [33,34]. Some cases of unilateral uveitis have been described [35]. Tularemia may be the cause of Parinaud oculoglandular syndrome (POGS), a unilateral granulomatous follicular palpebral or bulbar conjunctivitis associated with painful ipsilateral preauricular and submandibular lymphadenopathy [36]. Pharyngeal tularemia is the result of the ingestion of contaminated food or water. The predominant symptoms are fever, severe throat pain, and neck lymphadenopathy. Examination shows exudative pharyngitis and tonsillitis, one or more pharyngeal or tonsillar ulcers, and cervical lymphadenopathy [14]. Typhoidal tularemia is a form of tularemia where clinical presentation can range from acute sepsis to a chronic febrile illness. Patients do not have regional lymphadenopathy or some other localizing signs which can refer to other forms of tularemia. The most frequent symptoms are fever, chills, headache, anorexia, myalgias, sore throat, cough, nausea, vomiting, abdominal pain, and diarrhea, which is the main manifestation only in typhoidal tularemia. Patients can be dehydrated and hypotensive. Examination can show mild pharyngitis, cervical lymphadenopathy, and diffuse abdominal tenderness. Hepatomegaly and splenomegaly are found in later stages of illness [14,37]. Pneumonic tularemia, depending on the route of transmission, can be primary or secondary. Primary pneumonic tularemia results from breathing dusts or aerosols containing the organism. Some occupations present a risk of acquiring this form of tularemia, such as sheep shearers, farmers, landscapers, and laboratory workers. Symptoms include fever, cough with scant sputum production, substernal tightness, and pleuritic chest pain [38]. Secondary pneumonic tularemia results in bacterial spread through the bloodstream to the lungs and may be a complication of any of the major forms of tularemia but is most common with the typhoidal and ulceroglandular forms [31].

### 2.4. Diagnosis

The diagnosis of tularemia is based on clinical suspicion. If the patient has epidemiological risk factors, specific clinical features, or the symptoms developed after an arthropod, usually tick, bite, a diagnosis of tularemia should be suspected. Laboratory test results are nonspecific. Serology is most commonly used to confirm the diagnosis and should be used only in patients with a high degree of clinical suspicion of tularemia. It should not be used as a screening test. Techniques that are used for the detection of antibodies to *F. tularensis* are tube agglutination tests, microagglutination tests, latex agglutination tests, immunofluorescence assays (IFAs), enzyme-linked immunosorbent assay (ELISA), and immunochromatographic assays (ICTs). In Europe, ELISA is used more than in the United States, where tube agglutination and microagglutination tests are usually performed [39,40]. A serology test performed early may be negative during the early stage of the disease, so initial negative serology does not rule out infection with *F. tularensis*. So, it is important to take paired sera, with the first serum during the acute phase of illness (within the first week of onset) and the second serum 2–3 weeks later. Serological diagnosis of tularemia is confirmed by detecting the initial titer ≥ 160 for the tube agglutination test or ≥128 for the microagglutination tests. For definitive serologic diagnosis, a fourfold or greater change in titers of antibodies between acute and convalescent serum is necessary [41]. *F. tularensis* can be cultured from specimens like sputum, blood, pleural fluid, skin lesion drainage or biopsy, lymph node biopsy or drainage samples, and pharyngeal or ocular swabs. *F. tularensis* is rarely seen on Gram-stained smears or in tissue biopsy specimens. Cultures are often negative and the organism will not grow in routinely used cultures. It is important to notify laboratory personnel that tularemia is suspected so that appropriate media (modified Mueller–Hinton broth and thioglycollate broth) are used for cultivation, and also so that appropriate safety precautions are taken [14,42]. Polymerase chain reaction (PCR) is used for the rapid diagnosis of tularemia [43]. Other methods for the rapid diagnosis of tularemia have been developed, including direct fluorescent antibody (DFA) staining of clinical specimens and immunohistochemical staining of tissue, antigen detection in urine, specific monoclonal antibodies, and RNA hybridization with a 16S ribosomal probe (Table 2) [14].

### 2.5. Treatment

All patients with suspected or confirmed tularemia must be promptly treated with antibiotics. Aminoglycosides (gentamicin or streptomycin) are the drugs of choice for severe infections. The duration of aminoglycoside treatment is generally 7 to 14 days, depending on the severity of illness. Fluoroquinolone (ciprofloxacin) and doxycycline, as an alternative, are the drugs of choice for adults with mild or moderate infection. Tetracyclines as bacteriostatic agents are associated with a higher relapse rate after treatment, so they are recommended to be given for at least 14 days [44]. For tularemia meningitis, a successful treatment includes a combination of aminoglycoside with doxycycline or ciprofloxacin because penetration of the aminoglycosides into the cerebrospinal fluid is poor [45]. Treatment of pregnant patients may be challenging because the preferred antibiotic for the treatment of tularemia has potential risks to the fetus so optimal therapy for tularemia in pregnant patients is undefined. In one case report of tularemia in a pregnant woman from France, therapy with azithromycin was successful. Azithromycin may be an option for therapy in pregnant women in areas where infections caused by biovar 2 strains of *F. tularensis* subsp. *holarctica* do not occur because of their natural resistance to macrolides [46]. In another report of four pregnant patients, therapy with gentamicin and ciprofloxacin showed success [47]. The preferred antibiotic for the treatment of tularemia in children is gentamicin. It may be given intravenously and may have fewer adverse effects. The usual duration of therapy is 10 days. In cases of uncomplicated and mild disease, treatment can be shortened to five to seven days if there is an adequate clinical response. An appropriate alternative regimen for children with mild illness is oral ciprofloxacin. Doxycycline is not recommended for the treatment of tularemia in children [14]. Treatment of immunosuppressed patients with tularemia may be also challenging because they have an increased risk of treatment failure or relapse. In cases with enlarged lymph nodes, suppurate incision and drainage, or even excision of the lymph nodes, is warranted. In cases of pneumonic tularemia when empyema occurs, debridement and drainage are also warranted (Table 2).

### 2.6. Prevention

Currently, no vaccine against tularemia is available or approved for humans, so the main preventive measures involve minimizing exposure. These include avoiding contact with sick or dead animals, refraining from drinking or swimming in potentially contaminated water, using insect repellents, promptly removing ticks, properly cooking meats from wild animals, wearing masks, eye protection, and gloves when handling dead animals, and wearing clothing that covers exposed skin. Antibiotic prophylaxis is reserved for persons who have suspected or proven high-risk exposure to tularemia. This includes laboratory workers, autopsy personnel, persons exposed to *F. tularensis* in a bioterrorism event, and others exposed to materials contaminated with *F. tularensis* through nonintact skin and mucosal surfaces or aerosols. Prophylaxis is not indicated following a tick bite or after close contact with a patient with tularemia because person-to-person transmission does not occur. As prescribed by the doctor, antibiotics used for post-exposure prophylaxis in adults are oral ciprofloxacin 500 mg or doxycycline 100 mg, each taken twice daily for 14 days. Exposed children, other than in a bioterrorism event, may be observed for fever or other signs of illness without antibiotics. During a bioterrorist event, post-exposure prophylaxis in children is indicated. The recommended doses are ciprofloxacin 15 mg/kg orally twice daily (not to exceed 1 g daily) or doxycycline 2.2 mg/kg orally twice daily (for those < 45 kg) and 100 mg orally twice daily (for those ≥ 45 kg) [48].

## 3. Bubonic Plague

### 3.1. Microbiology and Epidemiology

Plague is a vector-borne zoonotic disease caused by the bacterium *Yersinia pestis*, which is an aerobic, Gram-negative coccobacillus in the *Yersiniaceae* family, order *Enterobacterales*. Plague is found in all continents except Oceania but most human cases since the 1990s have occurred in Africa. The Democratic Republic of Congo, Madagascar, and Peru are the three most endemic countries [49]. In Europe, in 2019, no cases of plague were reported by any of the EU/EEA countries, and plague has been absent from Europe for over half a century. However, it should be considered in the diagnosis for symptomatic travelers returning from risk areas because it is still widespread in the Americas, Africa, and Asia [50]. *Y. pestis* is usually found in small mammals and their fleas. It has been reported that more than 200 mammalian species have been infected with *Y. pestis*; some of them are squirrels, prairie dogs, rabbits, field mice, chipmunks, rats, bobcats, domestic cats, and camels, but rodents are the most important hosts [51]. The disease is transmitted between animals via fleas but it can also be transmitted from animals to humans directly [49]. Humans can be infected with *Y. pestis* via bites of rodent fleas (*Xenopsylla cheopis* and *Xenopsylla brasiliensis*), through direct contact with tissues and body fluids from infected animals, scratches, bites from infected domestic cats, by inhalation of cough droplets from a person or animal with pneumonic plague, or by exposure during examination from corpses and carcasses. In cases of pneumonic plague, the infected person can transmit plague to another person due to direct or close contact through cough droplets which contain bacteria. This is the only way the plague can spread between humans, which poses a danger of using *Y. pestis* as a potential bioterrorism agent that could cause pneumonic plague outbreaks [52,53] (Table 1).

### 3.2. Clinical Manifestation

The clinical characteristics of plague depend on the route of exposure. After 2 to 7 days, which is the usual incubation period, people infected with *Y. pestis* in most cases develop influenza-like symptoms. There are three main forms of plague: bubonic plague, septicemic plague, and pneumonic plague [54]. Other less common forms are pharyngeal and meningeal plague [55]. Bubonic plague is the most common clinical manifestation of *Y. pestis* infection and it accounts for 80 to 95 percent of cases [54]. This form usually results from the bite of an infected flea. During feeding, fleas regurgitate bacteria into the bite wound. Some of the inoculated bacteria are taken up by mononuclear cells and carried via lymphatics to the regional lymph nodes. The bacilli stimulate an intense inflammatory response in the lymph node and those which are draining the site of inoculation become painful, tender, and swollen and are referred to as “buboes” [56]. The most frequently involved are inguinal nodes, but axillary or cervical nodes may also be involved. Buboes vary in size from 1 to 10 cm. The overlying skin may be warm, erythematous, and edematous. Lymphadenopathy in bubonic plague is distinguishable from enlarged lymph nodes due to other causes by its sudden onset, their association with systemic signs of toxemia, and the absence of skin lesions or associated ascending lymphangitis. Occasionally, a small papule or scab demarcating the site of the flea bite can be seen due to careful examination. Rarely, eschars or ulcers can be seen on the site of inoculation, which can be confused with those of anthrax or tularemia [57]. Bubonic plague could become complicated by secondary pneumonia due to hematogenous spread of *Y. pestis*, which could enable airborne interhuman spread of the pathogen (Table 2).

### 3.3. Diagnosis

It is important to have a high index of suspicion for the diagnosis of bubonic plague. Early diagnosis leads to the early start of antibiotics, which is important to prevent severe complications leading to death. When bubonic plague is suspected, appropriate diagnostic specimens should be obtained, including blood cultures, bubo aspirates, and swabs of skin lesions. Bubo aspirates can be obtained by inserting a 20-gauge needle on a 10 mL syringe containing 2 mL of sterile saline solution into the bubo and withdrawing the plunger several times until the saline becomes blood-tinged. The gold standard for the diagnosis of plague is based on the isolation of *Y. pestis* by culture from clinical samples, which should be inoculated onto solid or liquid media (brain heart infusion broth, sheep blood agar, chocolate agar, or MacConkey agar) and held for 5 to 7 days. Bubo aspirate can be stained with Watson or Giemsa stain and Gram stain and then examined using light microscopy. Plague also can be confirmed by a passive hemagglutination test, the serological method for the detection of antibodies to the *Y. pestis* F1 antigen. This requires a four-fold rise in F1 antibody titers between acute and convalescent serum. Other rapid diagnostic methods for the diagnosis of plague are direct immunofluorescence assay (DFA) and PCR [54,58] (Table 2).

### 3.4. Treatment

Early recognition, diagnosis, and administration of appropriate antimicrobial therapy is essential because, if untreated, plague is fatal in over 50% of patients with bubonic plague and in nearly all cases of septicemic or pneumonic plague. Aminoglycosides are considered first-line treatment. Since the 1940s when streptomycin was introduced, it has been considered a first-line agent, but gentamicin has currently replaced it because streptomycin is no longer available in most countries, and it has been shown that gentamicin alone or in combination with tetracycline is an acceptable substitute [59]. Other alternatives are fluoroquinolones (levofloxacin, ciprofloxacin, or moxifloxacin) or doxycycline (Table 2).

### 3.5. Prevention

The best preventive measure in risk areas is to reduce exposure to wild rodents and their fleas. It is advisable to avoid handling dead animals, but if unavoidable, gloves should be worn. Additionally, close contact with suspected or confirmed plague cases in humans should be avoided. Other preventive measures include rodent and flea control, as well as the use of insect repellents. In healthcare settings, suspected cases of any form of plague should be placed in droplet precautions until pneumonia has been ruled out. In confirmed cases, droplet precautions should be continued until antibiotic therapy has been given for at least 48 h and there is evidence of clinical improvement. Standard precautions, including the use of personal protective equipment, should also be followed. Antibiotics can be used as prophylaxis in risky situations such as unprotected face-to-face contact with suspected or confirmed pneumonic plague cases. As prescribed by the doctor, antibiotics used for post-exposure prophylaxis are oral doxycycline 100 mg twice daily (for children 2.2 mg/kg twice daily, maximum 100 mg per dose), oral ciprofloxacin 500 to 750 mg twice daily (for children 15 mg/kg twice daily, maximum 750 mg per dose), oral levofloxacin 500 to 750 mg once daily (children < 50 kg 8 mg/kg twice daily, maximum 250 mg per dose), and oral moxifloxacin 400 mg once daily (for children, moxifloxacin dose depends on age). The duration of prophylaxis is typically seven days. There is currently no approved vaccine for plague [60,61,62].

## 4. Bartonellosis (*Bartonella henselae* Infection)

### 4.1. Microbiology and Epidemiology

The genus *Bartonella* (family *Bartonellaceae*; order *Hyphomicrobiales*) comprises Gram-negative intracellular bacteria transmitted by vectors and found in various mammalian hosts across the globe. Before 1990, only a single *Bartonella* species, *B. bacilliformis*, was officially recognized. Today, there are more than 36 known species, among which at least 20 have been linked to an ever-expanding range of diseases affecting both animals and humans [63,64]. Despite frequent characterizations of *Bartonella* species as newly emerging pathogens, it is actually a human pathogen from ancient times—*Bartonella quintana* has been discovered in the dental pulp of a human who lived over 4000 years ago [65]. Through history, the primary bacterial agents responsible for human diseases have been *Bartonella bacilliformis*, *Bartonella quintana*, and *Bartonella henselae*. While there are reports of other *Bartonella* species causing diseases in humans, their roles remain less clearly defined [66]. Molecular epidemiology methods have unveiled impressive diversity within the *Bartonella* genus. Numerous *Bartonella* species, each adapted to different mammalian host/s and transmitted by particular arthropod vectors, have been discovered over time. Infections caused by these species seem to be pervasive across various species and geographical areas. Infections caused by *B. quintana* and *B. henselae* are spread worldwide. South American bartonellosis, an infection caused by *B. bacilliformis*, is endemic to the Andean regions of Peru and Ecuador. An outbreak occurred in southern Colombia in the 1930s and 1940s, but no cases have been confirmed in the country since then. Additionally, sporadic cases have been rarely reported in Bolivia and Chile outside of endemic areas, which seem to refer to imported cases from returning travelers or migrants from endemic regions [67,68,69]. *Bartonella* spp. have been isolated from many hosts, including humans, cats, dogs, rodents, rabbits, horses, cattle, and other wild animals [63]. Usually, the infection is characterized by a persistent presence of bacteria within the erythrocytes of the reservoir. The infected blood is consumed by the blood-feeding arthropod and is passed on to another reservoir or an incidental host. The transmission cycle of bartonellosis usually involves various insect vectors, such as sand flies, cat fleas, and human body lice [63,70]. Although most *Bartonella* infections in humans develop after direct contact, such as a cat bite or scratch in the case of *B. henselae*, it can be transmitted via an insect vector, like body lice and fleas for *B. quintana* and fleas and sandflies for *B. bacilliformis* [71]. Emerging evidence indicates that *Bartonella* can also be transmitted by ticks, red ants, and spiders [64,66]. According to published case reports, *B. henselae* can also be transmitted by vectors (ticks), causing focal lymphadenitis in some patients [72,73,74,75] (Table 1).

### 4.2. Pathogenesis

In both humans and animals, *Bartonella* infections usually exhibit persistent bacteremia within red blood cells, often manifesting as a chronic or asymptomatic condition in their reservoir hosts. These bacteria have been known to infect various cells, including erythrocytes, endothelial cells, macrophages, and even human stem cells. The infection of erythrocytes is host-specific and is facilitated by the “Trw” type IV secretion system, which enables host-restricted adhesion to erythrocytes [76,77,78,79]. Localized tissue abnormalities can manifest in both reservoir and incidental hosts, with the proliferation of bacteria within vascular tissue potentially leading to the development of angio-proliferative tumors and inflammation [80,81]. Both animals and humans may experience conditions such as endocarditis, myocarditis, or various types of vascular disorders [63]. A secondary tissue phase of bartonellosis is associated with the occurrence of vasculo-proliferative lesions, exemplified by conditions like bacillary angiomatosis (caused by *B. henselae* and *B. quintana*) or verruga peruana (*B. bacilliformis*). These manifestations may also contribute to various other dermatological conditions [82,83]. The ability of *Bartonella* species to persist within immune-privileged intracellular environments is likely a pivotal factor in the establishment of chronic infections. Similar to other highly adapted intracellular pathogens transmitted by vectors, the precise determinants of disease presentation remain to be fully understood but are likely multifaceted. These determinants include variations in virulence among *Bartonella* species, disparities in the host’s immune response, and other contributing factors, such as coinfections, immunosuppression, concurrent noninfectious diseases, and malnutrition [63].

### 4.3. Clinical Manifestations

As cats are the natural reservoir for *B. henselae*, and the bacterium can cause intraerythrocytic bacteremia that may persist in cats for a year or longer [84], substantial evidence shows that *B. henselae* is the primary causative agent in most cat scratch disease (CSD) cases [85,86,87], which is a globally distributed illness [88]. In humans, *B. henselae* infiltrates endothelial cells and triggers an acute inflammatory response and the activation of a proinflammatory cascade [89]. CSD typically commences with a cutaneous lesion at the site of infection, called the primary inoculation lesion. Available data indicate that CSD can be contracted from a scratch or bite by a cat infected with *B. henselae* or through contact with cat fleas. Human transmission can also occur when cat saliva comes into contact with open skin or mucous membranes. A local lesion usually emerges 3 to 10 days after bacterial inoculation to the skin and typically progresses through vesicular, erythematous, and papular stages [90,91]. The hallmark of CSD is regional lymphadenopathy. However, regional lymphadenitis resembling CSD can develop after a tick bite [74,75]. Enlarged lymph nodes appear near the site of infection approximately two weeks (range: 7 to 60 days) after the bacterium enters the skin. In 85 to 90 percent of children, CSD presents as a localized skin and lymph node disease in the vicinity of the inoculation site [91]. However, in some cases, *Bartonella* spp. disseminate and affect the liver, spleen, eye, or the central nervous system. Involvement of visceral organs is a rare yet significant manifestation of CSD, especially in children. Visceral organ-related CSD can lead to persistent fever of unknown origin, abdominal discomfort, and/or weight loss [92,93,94]. Ocular presentations include Parinaud oculoglandular syndrome (POGS) [95]. The reason why some individuals with CSD experience localized infection while others develop disseminated disease remains unknown. Lymphadenopathy can persist for several months, and musculoskeletal symptoms, including myalgia, arthralgia, and arthritis, are observed sometimes, occurring in over 10 percent of patients [92,96]. Sometimes, lymphadenopathy can persist for a longer period of time. A case of a 6-year-old boy with a history of cervical lymphadenopathy which lasted for two years was reported [97]. Species-specific nested PCR for *B. henselae* in a whole blood sample was negative, but the amplification of an aliquot of a ten-day specific liquid culture detected *B. henselae* DNA. Neuroretinitis can be a manifestation of CSD, characterized by acute unilateral visual field loss due to optic nerve edema and star-shaped macular exudates [88]. In addition to typical ocular manifestations, patients may display symptoms such as isolated optic disc edema, branch retinal artery occlusion, and retinal infiltrations [98]. A case of an 11-year-old patient with binocular fundus nodular lesions has been reported [99]. Localized disease tends to be self-limiting, while disseminated disease can lead to life-threatening complications. A report of an immunocompetent individual who developed a *B. henselae* infection, which later advanced to hemophagocytic lymphohistiocytosis, necessitating immediate medical treatment, has recently been published [100]. CSD should be considered in the differential diagnosis of cases of unexplained fever or any lymphadenopathy syndrome [88] (Table 2).

### 4.4. Diagnosis

Diagnosing CSD relies on a combination of epidemiological, histological, and bacteriological criteria, as there is no single definitive standard. Common diagnostic methods for detecting *Bartonella* infection include serological testing, culture, histopathology, and polymerase chain reaction (PCR). Among the available blood tests, there are five options: Western blot, ELISA, IFA tests, PCR DNA detection, and culture. Due to the challenges in culturing *Bartonella* species (specific conditions and extended incubation periods), it is not routinely recommended [101,102]. Serology, particularly indirect fluorescent assay (IFA) or enzyme-linked immunosorbent assay (ELISA), serves as the preferred initial test. Differences between strains can result in false negative serology [103]. In a study involving 154 pediatric patients who were suspected of having CSD, the concerns related to the serological diagnosis (IFA) of CSD encompassed the utilization of low titers (1:128) for determining positivity, incomplete diagnostic assessment, and the absence of follow-up convalescent serologic testing [104]. Serologic tests (IFA and ELISA) for *B. henselae* were assessed on 51 Dutch patients with confirmed CSD (diagnosed through PCR confirmation). IgM assays demonstrated specificities of 93% (IFA) and 91% (ELISA) but had relatively low sensitivities (53% and 65%, respectively). In contrast, IgG testing, with specificities of 82% (IFA) and 91% (ELISA), displayed significantly higher sensitivity in IFA (67%) compared to ELISA (28%, *p* < 0.01) [105]. IFA IgG titers of 1:65 or 1:128 imply a possible *Bartonella* infection and it is suggested to repeat the test in 10–14 days. IgG titers of ≥1:256 strongly suggest active or recent infection. A positive IgM test strongly indicates an acute disease or a very recent infection, yet the production of IgM antibodies is typically short. There is notable cross-reactivity at the species level between *B. henselae* and *B. quintana*, particularly in IgG assays [106]. Histopathological examination of *Bartonella*-infected tissue typically reveals the presence of neutrophils, lymphocytes, and scattered debris within the lesions. The use of Warthin–Starry silver staining can reveal small, dark-staining bacteria, while electron microscopic analysis may show pleomorphic bacilli with a trilaminar wall. Advanced diagnostic techniques, such as PCR performed on lymph nodes or other materials, have been employed for *Bartonella* detection. PCR offers the advantages of high specificity and rapid identification, but it may lack sensitivity, ranging from 43% to 76%. Specifically, PCR sensitivity with lymph node tissue or aspirates is approximately 30–60% for CSD [101] (Table 2).

### 4.5. Treatment

In mild cases, treatment may be unnecessary. For these situations, supportive care, including antipyretics and anti-inflammatory medications, along with warm compresses applied to the injection site, may suffice. In instances of mild to moderate symptoms in immunocompetent patients, a course of azithromycin could be considered. Research has demonstrated that a 5-day course of azithromycin can alleviate severe lymphadenopathy pain but does not appear to reduce the overall duration of symptoms. As prescribed by the doctor, the recommended azithromycin dose is 10 mg/kg on the first day and 5 mg/kg from the second to the fifth day. Individuals weighing 45 kg or more can receive a dose of 500 mg on the first day and 250 mg from the second to the fifth day. Immunocompromised patients should undergo treatment to prevent the progression to severe systemic disease. There are various antibiotic regimens available, including doxycycline, rifampin, trimethoprim-sulfamethoxazole, and ciprofloxacin, for severe, disseminated disease. In severe cases of lymphadenitis, radical surgical intervention may be necessary, because the presence of suppuration can endure even after surgical incision and drainage [3,107] (Table 2).

## 5. Tick-Borne Lymphadenopathy (TIBOLA)

The rickettsiae are traditionally divided into three groups—the spotted fever group, the typhus group, and the scrub typhus group [108]. The spotted fever group accounts for most tick-borne rickettsioses. Tick-borne lymphadenopathy (TIBOLA) is a spotted fever group disease which is associated with a tick bite, an inoculation eschar on the scalp, and cervical lymphadenopathies. Raoult et al., in 1997, described a new tick-borne disease due to an infection with *Rickettsia slovaca* in a 39-year-old female patient who found a tick in her hair after taking an autumn walk in the woods of the Pyrenées mountains [109]. The tick was identified as *Dermacentor marginatus*, which in its adult form most often transmits *R. slovaca* to humans by bite during winter and early spring in a number of European countries [110]. Also in 1997, a total of 27 Hungarian patients were described with similar symptoms of enlargement of painful lymph nodes and eschar in the region of the tick bite, so based on that Lakos proposed the name TIBOLA [111]. In 2000, a Spanish study described similar clinical features in patients who were bitten by *D. marginatus*, and that syndrome was named Dermacentor-borne necrosis erythema and lymphadenopathy (DEBONEL) [112]. Except for eschar at the site of a tick bite and occipital and/or cervical lymphadenopathy, patients can rarely have fever, rash, headache, myalgia, and sometimes sequelae like persistent asthenia and localized alopecia at the eschar site [113] (Table 1).

### 5.1. Diagnosis

For serological diagnosis of TIBOLA, it is important to take convalescent-phase serum samples because, during the first week of illness, reactive antibodies are usually absent, so to confirm the diagnosis, a fourfold rise in the titer is necessary. Serological tests that can be used are indirect immunofluorescence (IFA), micro immunofluorescence (MIF) antibody test, enzyme-linked immunosorbent assay (ELISA), and Western blot immunoassay [114]. However, some authors claim that diagnosis of TIBOLA can usually be based on clinical and epidemiological features and, due to the low sensitivity and specificity of serology, if clinical features are typical, serological confirmation is not necessary [115]. The spotted fever group rickettsiae has minor antigenetic differences; therefore, serology tests are not able to discriminate members of this group but species-specific diagnoses can be made with polymerase chain reaction (PCR) amplification from blood, swab specimen of the eschar, skin biopsy samples, and other tissues. PCR of eschars and skin samples has higher sensitivity for detecting rickettsiae than PCR of whole blood [116,117] (Table 2).

### 5.2. Treatment

In patients with typical clinical and epidemiological features, it is important to start antibiotic treatment as soon as possible. As prescribed by the doctor, the drug of choice is doxycycline 100 mg twice daily for five to seven days. Doxycycline is also the preferred agent for the treatment of children. The dose of doxycycline for children who weigh ≤ 45 kg is 2.2 mg/kg twice per day (maximum daily dose 200 mg). New research shows the risk of dental staining with doxycycline is minimal when used in short courses [118,119,120] (Table 2).

## 6. Scrub Typhus

### 6.1. Epidemiology

Scrub typhus, an illness also known as bush typhus, is caused by the rickettsial bacterium *Orientia tsutsugamushi*. It spreads among people through bites from infected chiggers (larval mites) [121]. It has been estimated that *O. tsutsugamushi* causes illness in one million people each year. Scrub typhus is endemic in the Asia–Pacific region, known as the “tsutsugamushi triangle”, which includes Korea, China, Vietnam, Taiwan, Japan, Pakistan, India, Sri Lanka, Thailand, Malaysia, and the northern regions of Australia. In this region, it is one of the causes of fever of unknown origin. Additionally, some cases have been reported in Chile, Africa, and the Middle East, where serologic and molecular evidence of scrub typhus has been identified. This disease represents a serious public health problem [122,123,124]. Principal vectors for *O. tsutsugamushi* are trombiculid mites and other vertebrates (small mammals and birds), and humans are accidental hosts. After a bite, the bacterium multiplies at the site of infection and gradually induces local and systemic inflammatory response and manifestations of illness [125] (Table 1).

### 6.2. Clinical Manifestations

In patients suspected of having scrub typhus, finding an eschar at the bite site can be almost diagnostic. Patients with diagnosed scrub typhus usually have lymphadenopathy in the region of the primary affect (eschar) [126]. The prevalence of eschars is between 7 and 80%. In the beginning, they are a small papule that enlarges and turns black as central necrosis occurs. The common body sites of eschar are the groin, axilla, and waist. The common systemic signs of the disease are fever, gastrointestinal symptoms, malaise, cough, headache, and myalgia. At the end of the first week of illness, a maculopapular rash on the trunk spreading to the limbs can be seen. During the second week, some patients will experience systemic infection with different organ involvement (central nervous system, cardiovascular system, kidneys, lungs, and gastrointestinal system) [125] (Table 2).

### 6.3. Diagnosis

Regarding diagnostic methods, there is a lack of evidence on which test is the most appropriate in different clinical scenarios. Recent evidence shows that in early disease (first week), a molecular test (the quantitative PCR) from blood is the most sensitive. There are also serological tests in use, and both the IgM enzyme-linked immunosorbent assay and rapid diagnostic tests have excellent sensitivities and specificities [127] (Table 2).

### 6.4. Treatment and Prevention

Better outcomes can be achieved with early treatment [125]. As prescribed by the doctor, the first choice is doxycycline 200 mg orally once followed by 100 mg twice a day (for children < 40 kg it should be used in a dose of 2.2 mg/kg IV or p.o. twice daily) until the patient clinically improves, has been afebrile for 48 h, and has received treatment for a minimum of 7 days. An alternative is azithromycin in a dosage of 500 mg on the first day followed by 250 mg daily for 2 to 4 more days or 1 g initially, followed by 500 mg once daily for 2 days. Azithromycin remains the preferred drug during pregnancy or for children < 8 years. For children, azithromycin is used in the dose of 10 mg/kg/day for 5 days [128]. There is currently no available vaccine to prevent the transmission of scrub typhus, so prevention is based on avoiding exposure to mites (Table 2).

## 7. Other Rare Zoonotic Vector-Transmitted Causes of Lymphadenitis

In 2010, Dubourg et al. proposed the acronym “SENLAT” (scalp eschar and neck lymphadenopathy after tick bite) for human cases with this syndrome caused by other bacteria except *R. slovaca*, like other Rickettsia species (*Rickettsia raoultii*, *Rickettsia rioja*, and *Rickettsia massiliae*), *F. tularensis*, *B. henselae*, *B. burgdorferi*, and *Coxiella burnetii* [74,75,129,130]. One report described two patients who were infected with *R. slovaca* and also co-infected with *C. burnetii* [131]. *C. burnetii* causes Q fever, which can be asymptomatic, so they proposed that all patients infected with tick-borne pathogens be tested for *C. burnetii* infection [131]. *C. burnetii* has been detected in various ticks, but confirmed cases of human *C. burnetii* monoinfection via tick bites have not been reported [132].

In rare cases, lymphadenopathy can occur as part of clinical manifestations of Lyme disease. It is caused by *B. burgdorferi* sensu lato complex and is one of the most commonly diagnosed tick-borne infections across the globe. Ticks from the genus *Ixodes* are competent vectors for spirochetes [133] (Table 1). The condition progresses through three stages: early localized, early disseminated, and late. The majority of patients encounter symptoms during the early localized stage. Approximately 20% of patients progress to the early disseminated stage, which is usually characterized by multiple erythema migrans lesions, with lymphadenopathy as an additional possible manifestation [134]. The clinical symptoms resulting from the spread of spirochetes are typically associated with serological reactivity [135]. The serological diagnosis of Lyme borreliosis presently relies on a two-tier testing protocol. In this approach, positive and borderline outcomes in an initial sensitive screening assay undergo further examination for specificity using an immunoblot system [135]. It is important to emphasize that early manifestations of Lyme borreliosis, whether localized or disseminated, tend to resolve on their own without the need for antibiotic treatment. The primary objective in treating such patients is to expedite the resolution of symptoms and prevent subsequent complications [135]. For patients with erythema migrans (early or early disseminated disease–multiple erythema migrans), a 10-day course of doxycycline or a 14-day course of amoxicillin or cefuroxime axetil is recommended. For children aged 8 years or younger, the preferred antibiotic is amoxicillin or cefuroxime axetil [136] (Table 2).

In rare cases, focal vector-borne zoonotic lymphadenopathy can also be caused by parasites. Lymphatic filariasis, a neglected tropical disease, is an infection caused by nematodes of the *Filarioidea* family. The majority of cases globally are caused by *Wuchereria bancrofti.* However, in Asia the disease is also caused by *Brugia malayi* and *Brugia timori*. *B. malayi* may infect animals, so lymphatic filariasis caused by *B. malayi* is one of the zoonotic diseases that can be transmitted from animals to humans. For other filarial species that cause lymphatic filariasis, humans are the definitive hosts, and transmission occurs only between humans [137,138]. *B. malayi* is limited to Southern and Southeast Asia, mainly in China, India, Malaysia, the Philippines, Indonesia, some regions of Thailand, and parts of the Pacific [139,140]. The infection is spread by mosquito bites among humans. *Anopheles* and *Mansonia* are two genera of mosquitoes that are main vectors for *B. malayi* [141]. Animal reservoirs for *B. malayi* include domestic cats, dogs, primates, and pangolins [142]. *Anopheles* mosquitoes are found on every continent except Antarctica, while *Mansonia* mosquitoes are found in various tropical and subtropical regions worldwide. However, the presence of *B. malayi* and its vectors is most significant in Southeast Asian countries (Table 1). Since vectors of *B. malayi* are not limited only to Asia, there may be a risk of disease transmission for owners of imported infected animals from that part of the world. The mature worm lives in definitive host lymph vessels, engages in reproduction, and produces microfilariae. These small worms circulate in the bloodstream and transmit to mosquitoes during a bite. Within the mosquito, microfilariae undergo growth and maturation. Upon another mosquito bite, the larval worms move from the mosquito into the skin, traveling to the lymph vessels where they mature into adult worms, which lasts at least 6 months. The adult worms, which are usually found in lymph nodes in the inguinum and neck, have a lifespan of approximately 5–7 years, mate, and release millions of microfilariae into the bloodstream. Individuals carrying microfilariae can be potential sources of infection for others [139,140]. Lymphatic filariasis can have acute and/or chronic clinical manifestations but can also be asymptomatic. Acute manifestations include acute adenolymphangitis which presents with fever, painful lymphadenopathy, retrograde lymphangitis, and swelling of extremities. Repeated episodes of acute filarial infection can lead to chronic manifestations, which include lymphedema. In Brugian infections, this condition is typically limited to the distal extremities. Genital involvement is uncommon in Brugian filariasis. *B. malayi* infection is also associated with tropical pulmonary eosinophilia caused by an immune hyper-responsiveness to microfilariae in the lungs [143]. In addition to typical clinical manifestations and relevant epidemiological data, the diagnosis can be established by the detection of microfilariae under a microscope from blood samples. Blood should be collected during the night which coincides with the appearance of the microfilariae. Circulating filarial antigen (CFA) assays are not yet available for Brugian filariasis. Antifilarial antibody tests are serologic tests that detect elevated levels of IgG and IgG4, but they often have cross-reactivity with antigens from other helminths and cannot differentiate between active and past infections. Using ultrasound, adult worms can be seen in movement in the wide lymphatic vessels [139,144]. Diethylcarbamazine is the treatment of choice, taken orally for 1 or 12 days. Before treatment, physicians should check the patient for loiasis and onchocerciasis because this treatment can have serious side effects in these cases [139]. Doxycycline has shown efficacy in *B. malayi* infections. It exhibits both microfilaricidal and macrofilaricidal activity, so its addition could be appropriate. Also, in cases where diethylcarbamazine is contraindicated or not available, as prescribed by the doctor, doxycycline at a dose of 200 mg/day for four to six weeks can be used as an alternative first-line therapy [145] (Table 2).

Although described in humans occasionally, only after direct contact with infected animals, infection caused by *Corinebacterium pseudotuberculosis*, causing extensive lymphadenitis in animals, is readily transmitted among animals by arthropod vectors. As the vectors are shared between humans and animals, is it not clear if this type of transmission from animal to human does not exist, or if it has not been searched for in humans, but we feel that this possibility is worth mentioning in the context of this review.

*C. pseudotuberculosis* is a Gram-positive, facultative, intracellular bacterium which causes caseous lymphadenitis in animals, particularly sheep, goats, horses, and cattle. In the region of the peripheral lymph nodes, caseous lymphadenitis develops into chronic abscesses containing caseous pus. Infection occurs when *C. pseudotuberculosis* enters via skin wounds. Infection between animals is spread by vectors such as stable flies, horn flies, and house flies or by contact with an environment that is contaminated with exudate from abscesses [146,147]. Rarely, infection has been described in people who work closely with infected animals, like farmers and veterinarians. In humans, the disease manifests as granulomatous inflammation of axillary (due to infection through hands and arms), inguinal (source of infection may be environmental contamination), and cervical (due to ingestion of contaminated raw goat and cow milk as described in one case report) lymph nodes [148,149,150]. Two cases of pulmonary disease were described, both in veterinary students [151,152]. In a case report from Australia, the diagnosis of *C. pseudotuberculosis* infection was proven by microscopy and culture of pus specimens. Treatment includes excision of nodes, drainage, and antimicrobial treatment with β-lactam antibiotics, macrolides, or tetracyclines [153].

The zoonotic vector-borne causative agents of focal lymphadenitis, their reservoirs, vectors, and geographic distribution are summarized in Table 1, and their main clinical manifestations, diagnostic possibilities, and recommended therapy are described in Table 2.

## 8. Conclusions

In addition to common skin pathogens, acute focal lymphadenitis in humans can be caused by zoonotic pathogens in rare cases. Furthermore, it can develop in the absence of any direct or indirect contact with infected animals in cases when the microorganism is transmitted by an arthropod vector. A detailed epidemiological history and careful clinical examination, including a search for local bite wound or eschar, are crucial in pointing the differential diagnosis toward vector-borne zoonotic lymphadenitis. The spectrum of possible causative agents is broad; they can travel via infected people, animals, or vectors from endemic to nonendemic regions, and some of the involved microorganisms could have significant medical and public health impacts. This clinical entity is rare in most developed countries, and our aim was to raise awareness among clinicians, to describe possible clinical presentations, and present diagnostic methods according to various pathogens. When acute focal bacterial vector-borne zoonotic lymphadenitis is suspected, in severe or complicated cases, it seems prudent to apply combined aminoglycoside (or quinolone) plus doxycycline therapy as an empirical treatment, pending definite diagnostic results. In this field, the “one health approach” and further epidemiological and clinical studies are needed.

## Figures and Tables

**Table 1:** An overview of zoonotic vector-borne causative agents of focal lymphadenitis, their reservoirs, vectors, and geographic distributions.

Disease	Organism	Reservoirs and Vectors	Geographical Distribution
Tularemia	*F. tularensis* (divided into four subspecies): - *F. tularensis* subsp. *tularensis* (type A) - *F. tularensis* subsp. *holarctica* (type B) - *F. tularensis* subsp. *mediasciatica* - *F. tularensis* subsp. *novicida* *F. philomiragia* *F. hispaniensis* *F. opportunistica*	Reservoirs: Rabbits, beavers, muskrats, squirrels, voles, hares, hamsters, mice, rats, lemmings Vectors: Ticks, mosquitoes, biting flies, horse flies, fleas, lice	Worldwide in the Northern hemisphere
Bubonic plague	*Yersinia pestis*	Reservoirs: Most important: Rodents (found in 200 mammalian species) Vectors: Fleas	All continents except Oceania; since the 1990s most cases have occurred in Africa Three most endemic countries: Democratic Republic of Congo, Madagascar, and Peru
Bartonellosis (Cat scratch disease)	*Bartonella henselae*	Reservoirs: Cats (possible: other mammals) Vectors: Cat fleas (among cats) sand flies, human body lice. Possible: ticks, red ants, spiders	Worldwide
TIBOLA	*Rickettsia slovaca* *Rickettsia raoultii* *Rickettsia rioja* *Rickettsia massiliae*	Reservoirs: Ticks Vectors: Ticks (most often *Dermacentor marginatus*)	Worldwide
Borreliosis	*Borrelia burgdorferi* sensu lato complex	Reservoirs: White-footed mouse, chipmunks, voles, shrews, birds, squirrels, raccoons, skunks, shrews Vectors: Ticks (genus Ixodes)	Worldwide
Scrub typhus	*Orientia tsutsugamushi*	Reservoirs: Larval trombiculid mites (chiggers) Vectors: Larval trombiculid mites (chiggers)	Asia–Pacific region (endemic in Korea, China, Taiwan, Japan, Pakistan, India, Thailand, Laos, Malaysia, Vietnam, Sri Lanka, and Australia)
Malayan filariasis	*Brugia malayi*	Reservoirs: Domestic cats, dogs, primates, pangolins, humans Vectors: Mosquitos (main *Anopheles, Mansonia*)	Southern and Southeast Asia and parts of the Pacific

**Table 2:** The main clinical manifestations, recommended diagnostics, and therapy for zoonotic vector-borne causative agents of focal lymphadenitis.

Disease	Clinical Manifestations	Diagnosis	Therapy
Tularemia	Ulceroglandular tularemia: fever, skin lesion, and lymphadenopathy (cervical/occipital/inguinal) Glandular tularemia: regional lymphadenopathy without skin lesion Oculoglandular tularemia: eye pain, photophobia, increased lacrimation, sometimes lymphadenopathy Pharyngeal (oropharyngeal) tularemia: fever, severe throat pain, neck lymphadenopathy Pneumonic tularemia: fever, cough, pleuritic chest pain Typhoidal tularemia: sepsis or chronic febrile illness, without regional lymphadenopathy	Serology (most commonly used: ELISA, tube agglutination, and microagglutination tests) Culture (modified Mueller–Hinton broth and thioglycollate broth) Molecular testing–PCR DFA staining of clinical specimens and immunohistochemical staining of tissue	Mild or moderate disease: Adults: Doxycycline (100 mg p.o. BID for 14 to 21 days) or Ciprofloxacin (500 to 750 mg p.o. BID for 10 to 14 days) Children: Gentamicin (5 mg/kg IM or IV daily, divided every 8 or 12 h for 7 to 10 days) or Ciprofloxacin (20 to 40 mg/kg per day p.o. divided two doses for 10 to 14 days, maximum daily dose 1 g) Severe disease: Adults: Streptomycin (10 mg/kg IM BID for 7 to 10 days (max. daily dose 2 g) or Gentamicin (5 mg/kg IM or IV daily, divided every 8 h for 7 to 10 days Children: Streptomycin (30 to 40 mg/kg per day IM, in divided doses every 12 h for 7 to 10 days, maximum daily dose 2 g) or Gentamicin (5 mg/kg IM or IV daily, divided every 8 or 12 h for 7 to 10 days)
Bubonic plague	High fever, chills, weakness, headache, swelling of inguinal, axillary, or cervical lymph nodes, overlying skin may be warm and erythematous	Cultures of blood, bubo aspirates, swabs of skin lesions (brain heart infusion broth, sheep blood agar, chocolate agar or MacConkey agar) Microscopy evaluation of a bubo aspirate (Watson or Giemsa stain and Gram stain) Serology (passive hemagglutination test) DFA PCR	Adults: Gentamicin 5 mg/kg IM or IV QD Streptomycin 1 g IM or IV BID Ciprofloxacin 400 mg IV every 8 h; 750 mg p.o. BID Levofloxacin 750 mg IV, p.o. QD Moxifloxacin 400 mg IV, p.o. QD Doxycycline 200 mg loading dose, then 100 mg IV, p.o. BID Children: Gentamicin 4.5–7.5 mg/kg IM or IV QD Streptomycin 15 mg/kg IM or IV BID Ciprofloxacin IV: 10 mg/kg BID or TID (maximum 400 mg/dose). Oral: 15 mg/kg BID or TID (maximum 500 mg/dose every 8 h or 750 mg/dose every 12 h) Levofloxacin: <50 kg–8 mg/kg IV or p.o. BID (maximum 250 mg/dose) ≥50 kg–500 to 750 mg IV or p.o. BID Moxifloxacin: for pediatric patients with plague, other agents are recommended due to higher rates of QTc prolongation Doxycycline: <45 kg–4.4 mg/kg loading dose, then 2.2 mg/kg IV or p.o. BID ≥45 kg–200 mg loading dose, then 100 mg IV or p.o. BID
*Bartonella* *henselae* infection	CSD, regional granulomatous lymphadenitis Parinaud oculoglandular syndrome (atypical manifestation of CSD) Ocular manifestations of CSD: neuroretinitis, choroiditis, optic nerve granuloma, vascular-occlusive events FUO Endocarditis (patients with CHD or valvular abnormalities) Immunocompromised: BA, BP, bacteremia, endocarditis, FUO	Serological testing (IFA, ELISA) Culture (specific conditions and extended incubation—not routinely used) Histopathology PCR of tissue specimens or blood	Lymphadenitis: Adults: Azithromycin 500 mg on day 1 and then 250 mg for 4 days or Doxycycline 2 × 100 mg or Ciprofloxacin 2 × 500 mg or Trimethoprim-sulfamethoxazole 4 mg/kg orally (trimethoprim component) BID (max. 160 mg trimethoprim per dose) Children: Azithromycin < 45 kg 10 mg/kg orally on day 1, followed by 5 mg/kg orally for 4 days
TIBOLA	Eschar (typically on the scalp) and enlarged, often tender, cervical lymph nodes	Serologic tests: IFA, micro immunofluorescence (MIF) antibody test, ELISA, Western blot immunoassay PCR: from blood, swab specimen of the eschar, skin biopsy samples, and other tissues	Adults: Doxycycline 100 mg p.o. BID for five to seven days Children: Doxycycline ≤ 45 kg 2.2 mg/kg BID (maximum daily dose 200 mg) >45 kg should receive 100 mg BID
Borreliosis	Early localized or disseminated disease: Erythema migrans plus nonspecific clinical findings (e.g., fatigue, anorexia, headache, neck stiffness, myalgias, arthralgias, regional lymphadenopathy, fever)	In early localized illness: clinical presentation Serologic testing (two-tier testing protocol: screening assay and immunoblot for confirmation)	Adults: Doxycycline 100 mg p.o. BID for 10 days or Amoxicillin 500 mg p.o. TID for 14 days or Cefuroxime axetil 500 mg p.o. BID for 14 days Children (age 8 years or younger): Amoxicillin 50 mg/kg per day in three divided doses for 14 days or Cefuroxime axetil 30 mg/kg per day in two divided doses for 14 days
Scrub typhus	Acute febrile illness characterized by an eschar at the mite bite site, possible skin rash and other symptoms, which include localized and subsequent generalized lymphadenopathy, gastrointestinal symptoms, malaise, cough, headache and myalgia, and sometimes complications such as respiratory and renal failure, meningoencephalitis, and severe multiorgan failure	Serologic testing (IgM enzyme-linked immunosorbent assay and rapid diagnostic tests) Biopsy of an eschar or generalized rash PCR testing of blood samples Culture (available in only a few specialized laboratory centers)	Adults: Doxycycline 200 mg p.o. QD followed by 100 mg BID until the patient clinically improves, has been afebrile for 48 h, and has received treatment for a minimum of 7 days or Azithromycin 500 mg p.o. on the first day followed by 250 mg daily for 2 to 4 more days or 1 g initially, followed by 500 mg once daily for 2 days Children: Doxycycline 2.2 mg/kg IV or p.o. BID Azithromycin (for children < 8 years) 10 mg/kg/day for 5 days
Malayan filariasis	Acute lymphadenitis or lymphangitis, chronic lymphedema (elephantiasis), subcutaneous swelling, funiculo-epididymoorchitis, pulmonary eosinophilia, chyluria	Blood smears for microfilariae Ultrasound of lymphatic vessels Serology	Adults: Diethylcarbamazine 6 mg/kg/day as a single dose or in 3 divided doses for 1 or 12 days (14 to 21 days in patients with tropical pulmonary eosinophilia) or/plus Doxycycline 200 mg/day for 4–6 weeks Children: ≥18 months: 6 mg/kg p.o. as a single dose or 6 mg/kg/day in 3 divided doses for 12 days (14 to 21 days in patients with tropical pulmonary eosinophilia) Note: For patients with microfilaria in the blood, some clinicians recommend starting with a lower dosage with gradual increase over 3 days to 6 mg/kg/day in 3 divided doses on day 4 through the end of the treatment course

ELISA, enzyme-linked immunosorbent assay; PCR, polymerase chain reaction; DFA, direct immunofluorescence assay; BA, bacillary angiomatosis; BP, bacillary peliosis; CHD, congenital heart disease; CSD, cat-scratch disease; FUO, fever of unknown origin; IFA, indirect fluorescent assay; TIBOLA, tick-borne lymphadenopathy; QD, once daily; BID, two times a day; TID, three times a day.

## Data Availability

Not applicable.
